# Potential Adjunctive Effects of Quercetin–Curcumin Co-Supplementation in Adults with Mild-to-Moderate Long COVID: Results from a Pragmatic Exploratory Real-World Clinical Study

**DOI:** 10.3390/ph19081202

**Published:** 2026-07-31

**Authors:** Amjad Khan, Fazle Rabbani, Sami Ullah Mumtaz, Roha Javed, Ikram Ujjan, Ayesha Kanwal, Gabriele Conti

**Affiliations:** 1 Nuffield Division of Clinical Laboratory Sciences (NDCLS), Radcliffe Department of Medicine, University of Oxford, Oxford OX3 9DU, UK; 2 Department of Biochemistry, Liaquat University of Medical and Health Sciences (LUMHS), Jamshoro 76090, Pakistan; 3 Department of Psychiatry, Lady Reading Hospital (LRH), Peshawar 25000, Pakistan; 4 Department of Medicine, Narowal Medical College, Narowal 51600, Pakistan; 5 Department of Diet and Nutritional Sciences, University of Lahore, Lahore 54590, Pakistan; 6 Department of Pathology, Liaquat University of Medical and Health Sciences (LUMHS), Jamshoro 76090, Pakistan; 7 Human Microbiomics Unit, Department of Medical and Surgical Sciences, University of Bologna, Via Massarenti 9, 40138 Bologna, Italy

**Keywords:** Long COVID, polyphenols, quercetin, curcumin, supplements, complementary therapy, Nasafytol^®^

## Abstract

**Background/Objectives**: Long COVID is characterised by persistent symptoms such as fatigue, pain, cognitive impairment, sleep disturbances, and reduced quality of life (QoL) following SARS-CoV-2 infection. Proposed mechanisms include persistent immune activation, chronic inflammation, oxidative stress, endothelial dysfunction, mitochondrial disturbances, and virus-induced cellular senescence. Current management is largely supportive, highlighting the need for complementary strategies targeting these pathways. Natural polyphenols such as quercetin and curcumin possess anti-inflammatory, antioxidant, immunomodulatory, and potential senolytic properties that may modulate multiple pathways implicated in Long COVID. This study aimed to explore the potential complementary effects of oral quercetin–curcumin co-supplementation, administered alongside usual symptomatic management, in adults with persistent Long COVID symptoms. **Methods**: This single-centre, open-label, single-arm, pragmatic exploratory study enrolled 15 adults with Long COVID in an outpatient real-life clinical practice setting. Participants received a nutraceutical formulation containing quercetin (65 mg) and *Curcuma longa* extract standardised to provide 42 mg curcumin (Nasafytol^®^), administered as two capsules twice daily for 8 weeks in addition to standard care. The primary endpoint was change in overall symptom burden assessed using the COVID-19 Yorkshire Rehabilitation Scale (C19-YRS). Secondary outcomes included fatigue (Fatigue Severity Scale, FSS), pain (Brief Pain Inventory—Short Form, BPI-SF), cognitive function (Patient-Reported Outcomes Measurement Information System Cognitive Function Short Form 8a, PROMIS-CF-8a), mood (Hospital Anxiety and Depression Scale, HADS), sleep quality (Pittsburgh Sleep Quality Index, PSQI), autonomic symptoms (Composite Autonomic Symptom Score-31, COMPASS-31), and health-related QoL (Medical Outcomes Study 36-Item Short-Form Health Survey, SF-36). **Results**: After 8 weeks, overall symptom burden significantly decreased (C19-YRS median 50 to 32; *q* = 0.018). Significant reductions were observed in patient-reported fatigue (*q* = 0.006), pain severity and interference (*q* = 0.006), and improved physical health-related QoL (SF-36 PCS; *q* = 0.025). Numerically favourable changes were also observed in cognitive function, anxiety, sleep quality, and autonomic symptoms, although these did not reach statistical significance. The supplementation was generally well tolerated with no serious adverse events. **Conclusions**: Quercetin–curcumin co-supplementation was associated with lower patient-reported symptom burden, fatigue, and pain and better physical health-related QoL after 8 weeks of supplementation in adults with Long COVID. These observed findings represent treatment-associated changes within this uncontrolled exploratory study and should be interpreted cautiously. Further evaluation in adequately powered randomised placebo-controlled trials is warranted. Trial registration: ClinicalTrials.gov (ID NCT06974058).

## 1. Introduction

Long COVID, also referred to as post-COVID condition, is a multisystem disorder characterised by persistent, relapsing, or newly emerging symptoms that usually occur within three months of acute SARS-CoV-2 infection, last for at least two months, and cannot be explained by an alternative diagnosis [1]. It has emerged as an important public health challenge, affecting a substantial proportion of individuals recovering from COVID-19 and causing prolonged impairment in physical functioning, cognitive performance, psychological well-being, and health-related quality of life (QoL) [1,2,3,4,5]. The clinical presentation is heterogeneous and commonly includes fatigue, cognitive dysfunction (“brain fog”), musculoskeletal pain, dyspnoea, sleep disturbances, autonomic dysfunction, and neuropsychological symptoms, many of which may persist for months after the initial infection [1,2,3,4,5].

Although the pathophysiology of Long COVID remains incompletely understood, increasing evidence suggests that persistent immune dysregulation, chronic inflammation, oxidative stress, endothelial and microvascular dysfunction, mitochondrial impairment, dysautonomia, and virus-induced cellular senescence contribute to the persistence of symptoms following acute SARS-CoV-2 infection [1,2,3,4,5]. Collectively, these interconnected mechanisms provide a biological rationale for exploring adjunctive interventions capable of simultaneously targeting multiple pathogenic pathways.

Despite the growing recognition of Long COVID, effective pharmacological treatment options remain limited. Current management is largely supportive and symptom-directed, including rehabilitation programmes, psychological support, and management of organ-specific complications [2,3,5,6,7]. Consequently, there is considerable interest in identifying safe adjunctive therapies capable of modulating the biological mechanisms implicated in persistent post-COVID symptoms [1,2,3,4,5].

Among the adjunctive therapeutic strategies being investigated, naturally occurring polyphenols have attracted increasing attention because of their generally favourable safety profile and their antioxidant, anti-inflammatory, immunomodulatory, and endothelial-protective properties. Among these, quercetin and curcumin (*Curcuma longa* L. (Zingiberaceae)) are of particular interest because they simultaneously modulate multiple biological pathways implicated in both acute and Long COVID [8,9,10,11,12,13,14,15,16,17,18,19,20]. Randomised clinical trials have shown that, when administered alongside standard care, supplementation with quercetin or curcumin individually was associated with improvements in clinical outcomes and inflammatory markers [8,9,10,11,12,13,14,15,16,17,18,19,20], while adjunctive supplementation with the combined quercetin–curcumin formulation was associated with faster symptom resolution, earlier viral clearance, and improved clinical recovery in patients with mild-to-moderate COVID-19 managed in outpatient settings [21,22]. Similar benefits, including earlier hospital discharge and improved clinical outcomes, have also been reported in hospitalised patients receiving the same formulation alongside standard care [23]. Collectively, these clinical findings suggest that modulation of multiple biological pathways may contribute to improved recovery following SARS-CoV-2 infection.

The clinical benefits observed with quercetin, curcumin, and their combination are biologically plausible given their pleiotropic biological activities and complementary mechanisms of action. Experimental studies have demonstrated that both compounds individually modulate multiple biological pathways implicated in COVID-19 pathophysiology, including nuclear factor kappa-light-chain-enhancer of activated B cells (NF-κB), mitogen-activated protein kinase (MAPK), and NOD-like receptor family pyrin domain-containing 3 (NLRP3) inflammasome signalling, oxidative stress, endothelial dysfunction, and the production of pro-inflammatory cytokines such as interleukin-6 (IL-6), tumour necrosis factor-α (TNF-α), and interleukin-1β (IL-1β) [8,9,10,11,12,13,14,15,16,17,18,19,20]. Emerging evidence also suggests that quercetin possesses senolytic activity, providing a potential mechanism for attenuating virus-induced cellular senescence and its associated pro-inflammatory secretory phenotype [13]. Although both polyphenols influence many of the same biological processes implicated in COVID-19, their mechanisms are only partially overlapping, with each compound modulating distinct as well as shared molecular targets. Consequently, co-supplementation has the potential to broaden modulation of interconnected pathogenic processes through their complementary biological activities. Given the complex and multifactorial nature of Long COVID, simultaneously targeting these complementary pathways provides a strong biological rationale for evaluating quercetin–curcumin co-supplementation in patients with persistent post-COVID symptoms.

However, despite the biological rationale and encouraging evidence from acute COVID-19, clinical evidence evaluating quercetin–curcumin supplementation in individuals with established Long COVID remains lacking. Given the overlap between the mechanisms implicated in acute COVID-19 and those proposed to sustain persistent post-COVID symptoms, it is plausible that this combination may provide benefit beyond the acute phase of infection. Nevertheless, to the best of our knowledge, this hypothesis has not previously been evaluated in a prospective clinical study involving individuals with Long COVID.

Therefore, the present pragmatic exploratory clinical study aimed to evaluate the potential adjunctive effects of oral quercetin–curcumin co-supplementation, administered alongside usual symptomatic management, in adults with persistent Long COVID. Specifically, we investigated changes in overall symptom burden, fatigue, pain, cognitive function, mood, sleep quality, autonomic symptoms, and health-related QoL following eight weeks of supplementation. As an exploratory single-arm study, the primary objective was to generate preliminary clinical evidence and estimate the magnitude of potential treatment-associated changes to inform the design of future adequately powered randomised controlled trials.

## 2. Results

**Figure 1:**
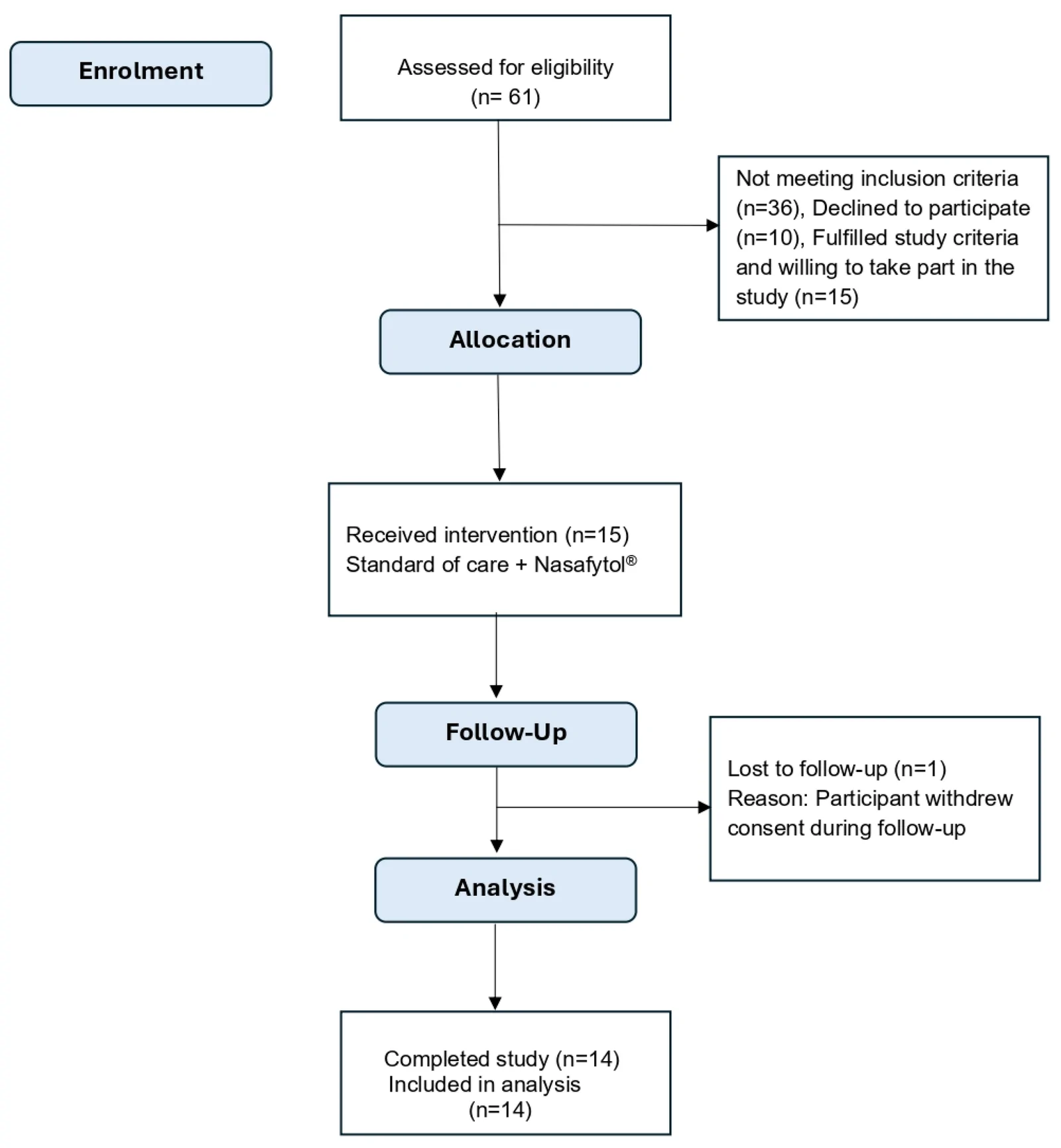
CONSORT-style participant flow diagram.

Baseline demographic and clinical characteristics of the study population are summarised in Table 1. A total of 15 adults with persistent Long COVID symptoms were enrolled in this pragmatic exploratory study (Figure 1). Of these, 14 participants completed the study, whereas one participant withdrew during follow-up at their own discretion. The median age of the study population was 37.00 (IQR 20.00) years, and the median body mass index (BMI) was 24.60 (IQR 10.35) kg/m^2^. Baseline vital signs were within physiological ranges, including systolic and diastolic blood pressure, heart rate, respiratory rate, and body temperature. Acute SARS-CoV-2 infection occurred predominantly in 2021 (67%), followed by 2022 (27%) and 2023 (7%). Most participants experienced a non-severe acute disease course, with 93% managed in a home-based setting and only one participant requiring hospitalisation. Long COVID symptoms developed primarily between 3 and 6 months after acute infection, with a relatively even distribution within this interval. At baseline, two-thirds of participants reported no relevant comorbidities, whereas approximately one-third had at least one pre-existing medical condition. The majority of participants (93%) were fully vaccinated against COVID-19 at the time of enrolment.

### 2.1. Changes in Overall Symptom Burden

**Figure 2:**
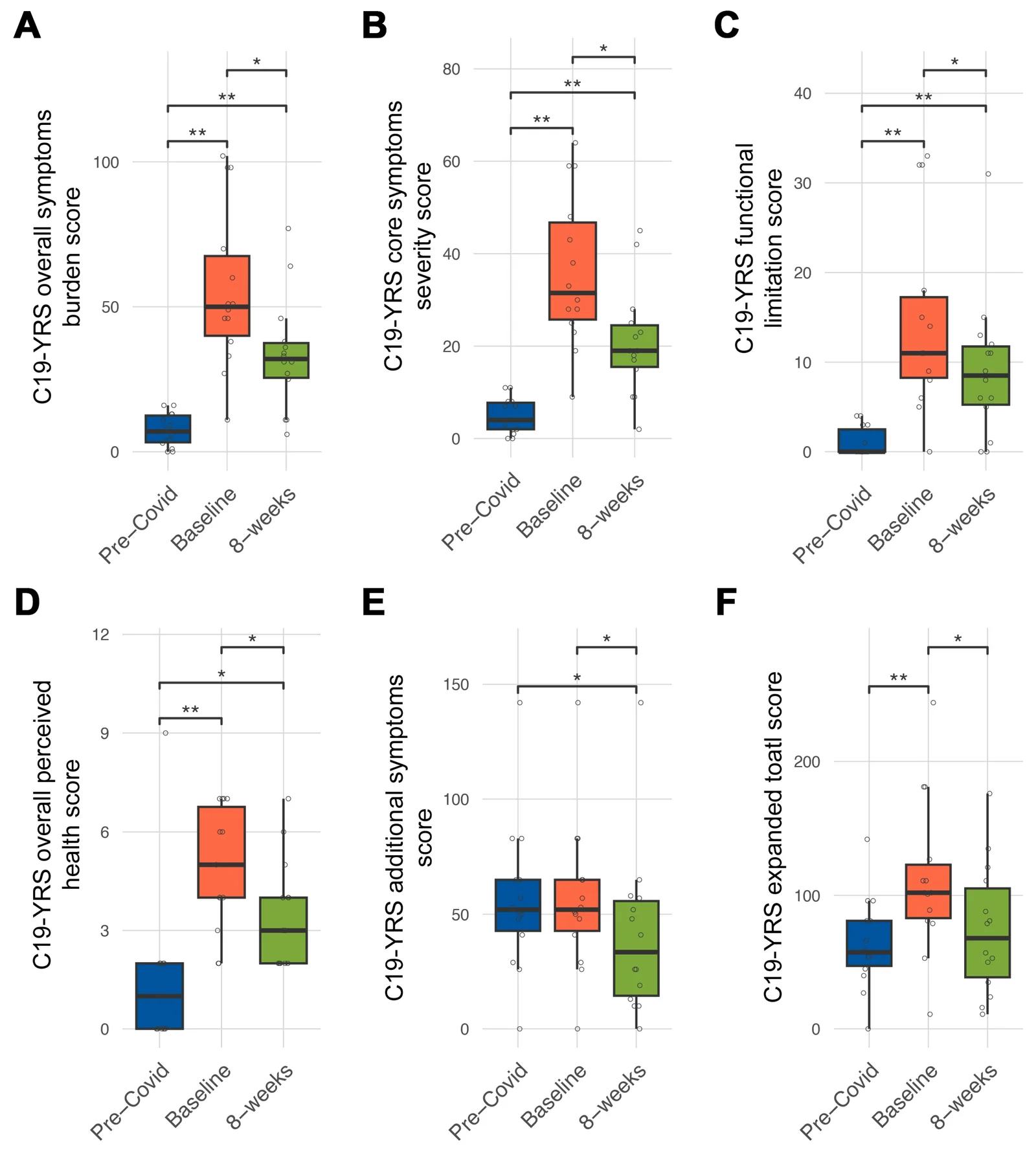
Longitudinal changes in Long COVID symptom burden assessed using the COVID-19 Yorkshire Rehabilitation Scale (C19-YRS) following 8 weeks of supplementation. Panels show (**A**) total symptom burden score, (**B**) symptom severity score, (**C**) functional limitation score, (**D**) overall perceived health score, (**E**) additional symptom score, and (**F**) expanded total score. Boxplots show retrospectively self-reported pre-COVID, baseline, and Week 8 scores. Boxes represent the interquartile range (IQR), the centre line indicates the median, whiskers extend to 1.5 × IQR, and points represent individual participants. Wilcoxon signed-rank tests with FDR correction were used for paired comparisons. Pre-COVID assessments represent retrospective self-reported estimates and are provided for exploratory contextual comparison only. * *q* ≤ 0.05; ** *q* ≤ 0.01.

At baseline, participants exhibited a substantially higher symptom burden compared with their self-reported pre-COVID status across all major domains of the COVID-19 Yorkshire Rehabilitation Scale (C19-YRS) (Wilcoxon paired test FDR-adjusted, *q* ≤ 0.003, *r* ≥ 0.8), confirming the persistence and clinical relevance of Long COVID symptoms (Figure 2). These retrospective pre-COVID comparisons were based on participant self-reported recall and are therefore exploratory and contextual in nature rather than formal longitudinal comparator assessments. Following 8 weeks of supplementation, participants reported lower overall symptom burden scores compared with baseline. The total C19-YRS score decreased from a median (IQR) of 50 (27.5) at baseline to 32 (12) at Week 8 (*q* = 0.018, *r* = 0.78) (Figure 2A), corresponding to an approximate 36% reduction in global symptom severity.

Consistent reductions were observed across individual C19-YRS domain scores (*q* ≤ 0.023, *r* ≥ 0.67). Symptom severity scores decreased from 31.5 (21) to 19 (9) (*q* = 0.018) (Figure 2B), while functional limitation scores decreased from 11 (9) to 8.5 (6.5) (*q* = 0.023) (Figure 2C). Participants also reported an improvement in overall perceived health status, with scores decreasing from 5 (2.75) to 3 (2) (*q* = 0.023) (Figure 2D).

Similar reductions were observed in additional symptom domains, with both the additional symptom score and the expanded total score showing significant reductions at 8 weeks (52 (22.25) vs. 33.5 (41.25), *q* = 0.023 (Figure 2E), and 102 (40) vs. 68 (66.5), *q* = 0.018 (Figure 2F), respectively).

Collectively, these findings demonstrate a lower patient-reported Long COVID symptom burden at Week 8 compared with baseline.

### 2.2. Changes in Fatigue

**Figure 3:**
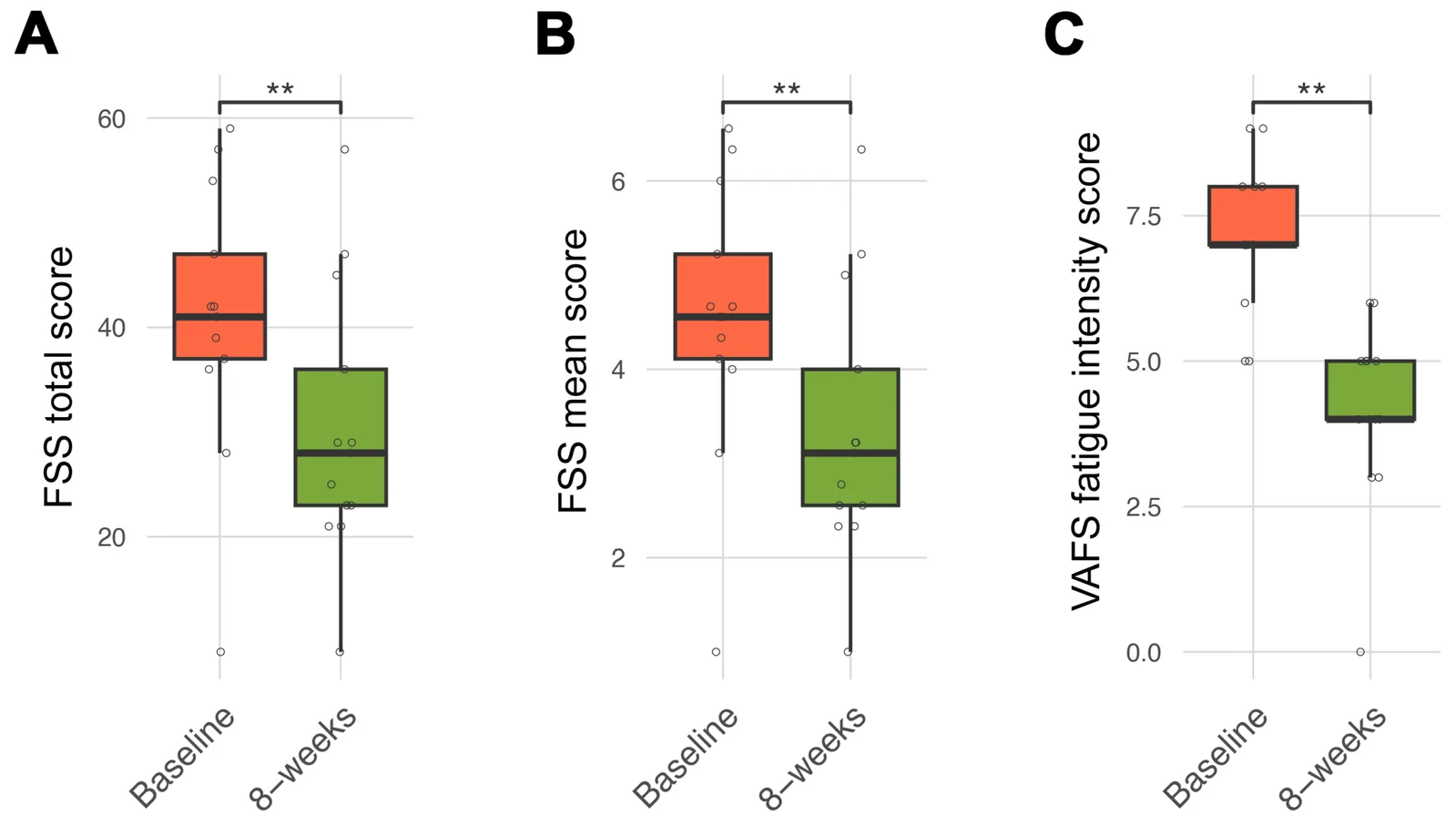
Longitudinal changes in fatigue-related outcomes following 8 weeks of supplementation. Panels show (**A**) Fatigue Severity Scale (FSS) total score, (**B**) FSS mean score, and (**C**) Visual Analogue Fatigue Scale (VAFS) fatigue intensity score. Boxes represent the interquartile range (IQR), the centre line indicates the median, whiskers extend to 1.5 × IQR, and points represent individual participants. Wilcoxon signed-rank tests with FDR correction were used for paired comparisons. ** *q* ≤ 0.01.

Lower fatigue scores were observed at Week 8 compared with baseline, as assessed using the Fatigue Severity Scale (FSS), and they were consistently observed across multiple fatigue-specific assessment tools (Figure 3). The total FSS score decreased from a median (IQR) of 41 (10) at baseline to 28 (13) at Week 8 (*q* = 0.006, *r* = 0.83), corresponding to a reduction in overall fatigue burden (Figure 3A). Consistent findings were observed when evaluating the mean FSS score, which declined from 4.56 (1.11) at baseline to 3.11 (1.44) at 8 weeks (*q* = 0.006), corresponding to lower fatigue severity scores (Figure 3B). Fatigue intensity assessed using the Visual Analogue Fatigue Scale (VAFS) also declined significantly, from 7 (1) to 4 (1) (*q* = 0.006), corresponding to an approximate 43% reduction in perceived fatigue severity (Figure 3C). Overall, lower fatigue scores were consistently observed across the different fatigue assessment instruments.

### 2.3. Changes in Pain-Related Outcomes

**Figure 4:**
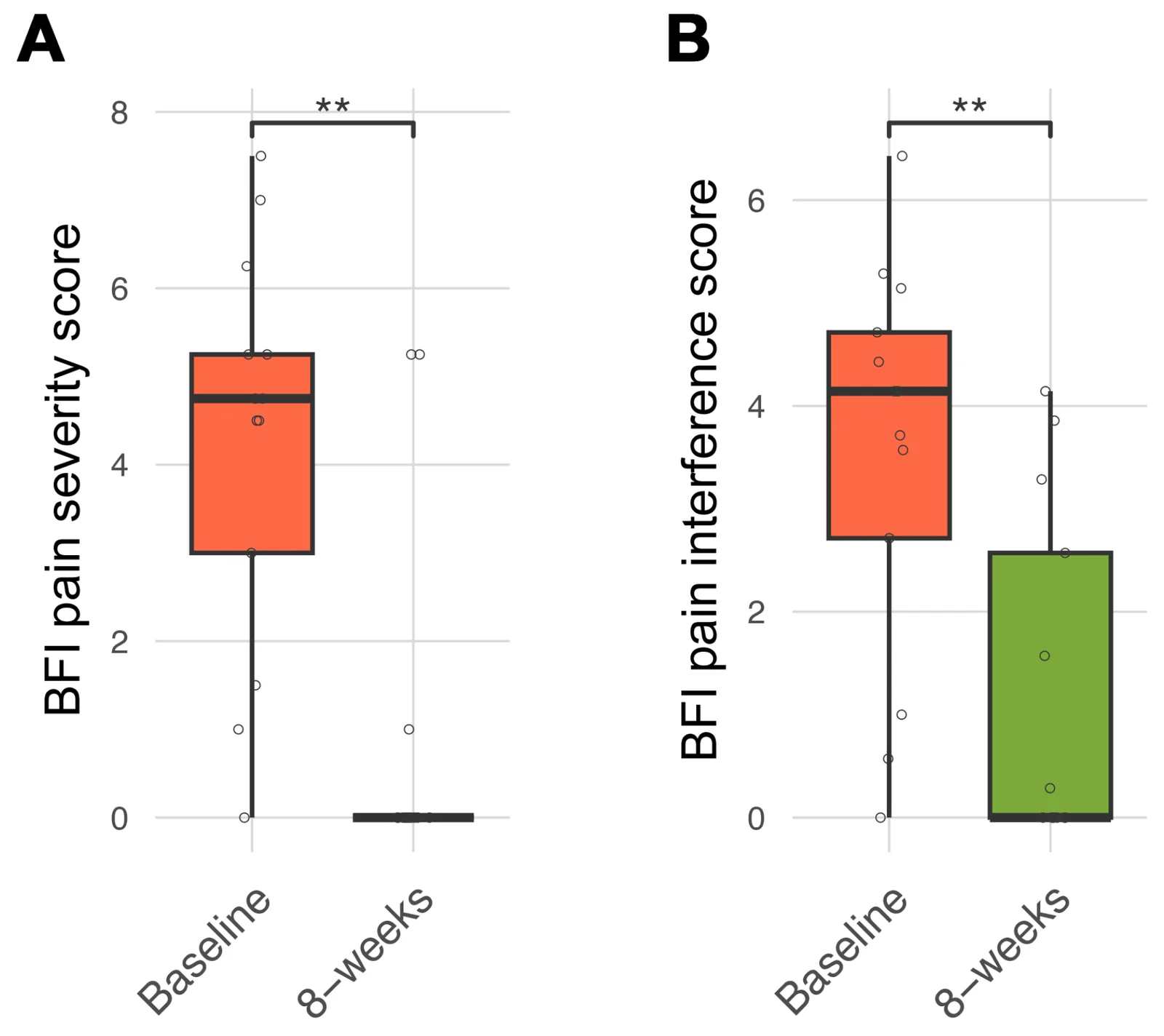
Longitudinal changes in pain-related outcomes assessed using the self-reported Brief Pain Inventory—Short Form (BPI-SF) following 8 weeks of supplementation. Panels show (**A**) pain severity score and (**B**) pain interference score. Boxes represent the interquartile range (IQR), the centre line indicates the median, whiskers extend to 1.5 × IQR, and points represent individual participants. Wilcoxon signed-rank tests with FDR correction were used for paired comparisons. ** *q* ≤ 0.01.

Lower pain-related scores were observed at Week 8 compared with baseline, as assessed using the self-reported Brief Pain Inventory—Short Form (BPI-SF) (Figure 4). Both pain severity and pain interference scores declined over the intervention period. The Pain Severity (PS) score decreased from a median (IQR) of 4.75 (2.25) at baseline to 0 (0) at Week 8 (*q* = 0.006, *r* = 0.86), corresponding to lower pain severity scores (Figure 4A). Similarly, the Pain Interference (PI) score decreased from 4.14 (2) at baseline to 0 (2.57) at 8 weeks (*q* = 0.006, *r* = 0.86), reflecting a reduced impact of pain on daily functioning (Figure 4B). Overall, lower pain-related scores were consistently observed across both BPI-SF domains.

### 2.4. Changes in Cognitive Function, Emotional Well-Being, and Sleep Quality

**Figure 5:**
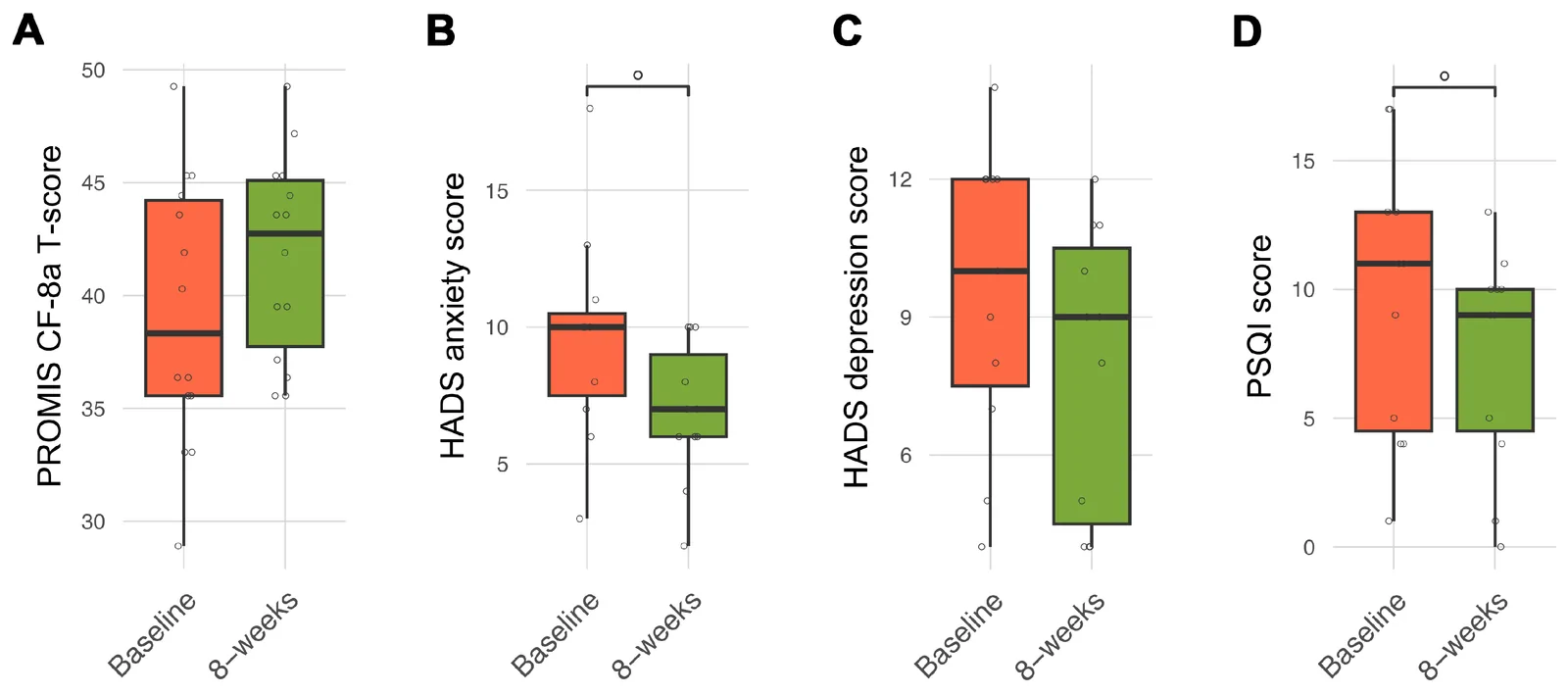
Cognitive function, emotional well-being, and sleep quality following 8 weeks of supplementation. Panels show (**A**) PROMIS Cognitive Function—Short Form 8a (PROMIS-CF-8a) T-score, (**B**) Hospital Anxiety and Depression Scale—Anxiety Subscale (HADS-A), (**C**) Hospital Anxiety and Depression Scale—Depression Subscale (HADS-D), and (**D**) Pittsburgh Sleep Quality Index (PSQI) global score. Boxes represent the interquartile range (IQR), the centre line indicates the median, whiskers extend to 1.5 × IQR, and points represent individual participants. Wilcoxon signed-rank tests with FDR correction were used for paired comparisons. ° *q* ≤ 0.1 indicates a statistical trend.

Cognitive function, assessed using the Patient-Reported Outcomes Measurement Information System Cognitive Function Short Form 8a (PROMIS-CF-8a), showed numerically higher scores over the 8-week intervention period, although changes did not reach statistical significance. PROMIS-CF-8a T-scores increased from a median (IQR) of 38.33 (8.66) at baseline to 42.74 (7.35) at Week 8 (*q* = 0.15, *r* = 0.4), consistent with better perceived cognitive functioning (Figure 5A). Measurement reliability remained stable over time, as reflected by consistent standard error estimates (median (IQR): 2.21 (0.07) vs. 2.20 (0.07)).

Emotional well-being demonstrated differential patterns across anxiety and depressive symptoms (Figure 5B,C). Anxiety levels assessed using the Hospital Anxiety and Depression Scale—Anxiety Subscale (HADS-A) decreased from 10 (3) at baseline to 7 (3) at 8 weeks (*q* = 0.085, *r* = 0.72), consistent with lower anxiety scores. Depressive symptoms assessed using the HADS—Depression Subscale (HADS-D) also decreased numerically from 10 (4.5) to 9 (6) (*q* = 0.11, *r* = 0.55) (Figure 5C), although this change did not reach statistical significance.

Sleep quality, evaluated using the Pittsburgh Sleep Quality Index (PSQI), also showed numerically lower scores (Figure 5D). Median PSQI scores decreased from 11 (9) at baseline to 9 (5) at 8 weeks (*q* = 0.085, *r* = 0.54), consistent with lower self-reported sleep disturbance scores, although the difference was not statistically significant.

Overall, numerically lower anxiety, depressive symptom, and sleep disturbance scores, together with higher PROMIS-CF-8a scores, were observed at Week 8 compared with baseline, although not all changes reached statistical significance.

### 2.5. Changes in Autonomic Dysfunction

**Figure 6:**
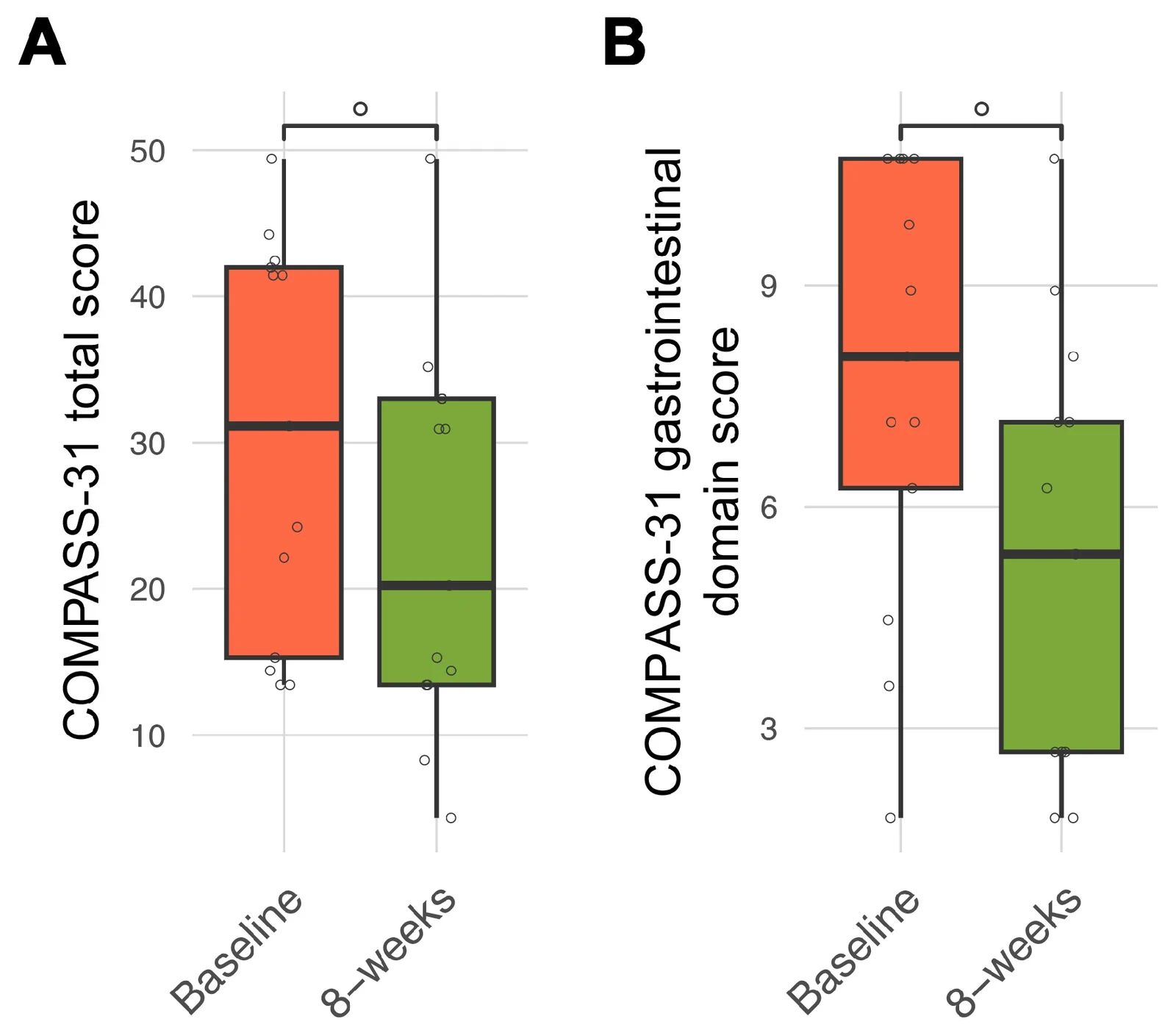
Longitudinal changes in autonomic dysfunction assessed using the self-reported Composite Autonomic Symptom Score (COMPASS-31) following 8 weeks of supplementation. Panels show (**A**) COMPASS-31 total score and (**B**) COMPASS-31 gastrointestinal domain score. Boxes represent the interquartile range (IQR), the centre line indicates the median, whiskers extend to 1.5 × IQR, and points represent individual participants. Wilcoxon signed-rank tests with FDR correction were used for paired comparisons. ° *q* ≤ 0.1 indicates a statistical trend.

Autonomic symptoms, assessed using the self-reported Composite Autonomic Symptom Score-31 (COMPASS-31), demonstrated a trend toward improvement over the 8-week intervention period. The total COMPASS-31 score decreased from a median (IQR) of 31.12 (26.67) at baseline to 20.22 (19.57) at Week 8 (*q* = 0.063, *r* = 0.8), suggesting a reduction in dysautonomia-related symptom burden (Figure 6A). Domain-specific analyses indicated that this numerical change was primarily driven by the gastrointestinal domain, which decreased from 8.04 (4.46) at baseline to 5.36 (4.46) at 8 weeks (*q* = 0.077) (Figure 6B). No statistically significant changes were observed in the remaining COMPASS-31 domains, including orthostatic intolerance, vasomotor, secretomotor, bladder, and pupillomotor symptoms (Figure S1).

### 2.6. Changes in Health-Related Quality of Life

**Figure 7:**
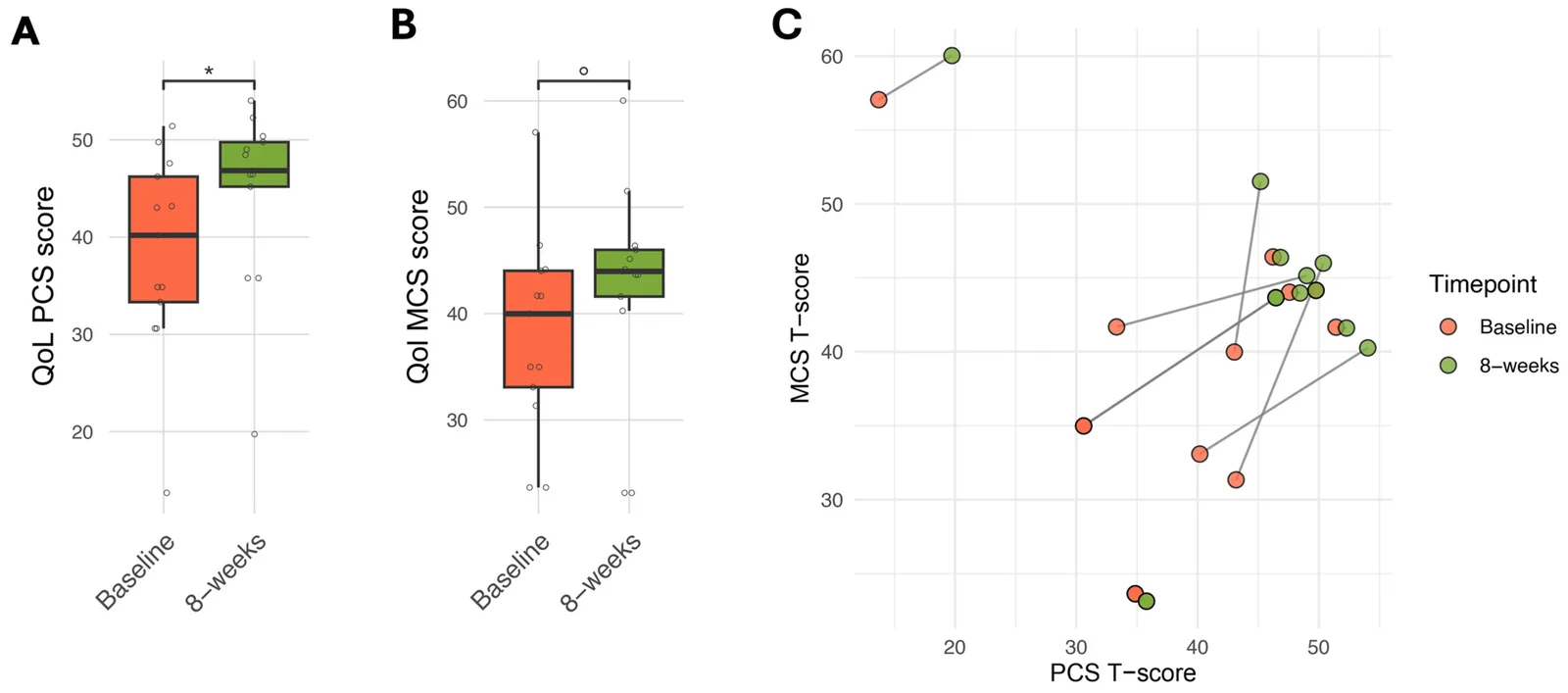
Changes in health-related quality of life following 8 weeks of Nasafytol^®^ supplementation. Panels show (**A**) SF-36 Physical Component Summary (PCS) T-score, (**B**) SF-36 Mental Component Summary (MCS) T-score, and (**C**) individual-level changes in the combined PCS–MCS profile between baseline and Week 8. For boxplots, boxes represent the interquartile range (IQR), the centre line indicates the median, whiskers extend to 1.5 × IQR, and points represent individual participants. Wilcoxon signed-rank tests with FDR correction were used for paired comparisons. Overall changes in the combined PCS–MCS profile were explored using PERMANOVA (Euclidean distance, 9999 permutations). * *q* ≤ 0.05; ° *q* ≤ 0.1.

Health-related QoL was assessed using the Medical Outcomes Study 36-Item Short-Form Health Survey (SF-36) to evaluate changes in physical and mental well-being following the intervention (Figure 7).

The Physical Component Summary (PCS) score increased from a median (IQR) of 40.18 (12.90) at baseline to 46.84 (4.57) at Week 8 (*q* = 0.025, *r* = 0.87) (Figure 7A), corresponding to higher physical health-related QoL scores.

The Mental Component Summary (MCS) score also increased from 39.98 (10.95) to 43.97 (4.39) over the intervention period (Figure 7B); however, this change did not reach statistical significance (*q* = 0.093, *r* = 0.49).

To evaluate the combined QoL profile, an exploratory Permutational Multivariate Analysis of Variance (PERMANOVA) based on Euclidean distances was performed. This analysis suggested a difference in the combined PCS–MCS profile between baseline and 8 weeks (R^2^ = 0.076, *p* = 0.0005) (Figure 7C). Given the exploratory study design and the very small sample size, this finding should be regarded as exploratory and interpreted cautiously and considered supportive of the univariate PCS and MCS results rather than independently establishing a global treatment effect.

### 2.7. Safety, Tolerability, and Adherence

Overall, the supplementation was well tolerated throughout the 8-week intervention period. No serious adverse events were reported, and no participants discontinued the study due to adverse effects. Adherence to the supplementation regimen was high based on participant self-report, review of supplement use, and weekly follow-up contacts. Adherence assessment was additionally supported by a review of supplement use/returned supplement packaging, and overall adherence to supplementation was estimated to be greater than 90% across the study population.

Three participants reported mild and transient gastrointestinal symptoms during the early phase of supplementation, including abdominal discomfort and mild gastrointestinal upset. These symptoms were clinically assessed by the investigators as possibly related to supplementation. These symptoms were self-limited, resolved spontaneously without medical intervention, and did not require discontinuation or dose adjustment of the study supplement.

Adverse events and tolerability were assessed through participant self-report during weekly follow-up contacts and at the Week 8 assessment visit. Adverse events were evaluated clinically based on symptom severity, duration, need for medical intervention, and suspected relationship to supplementation; however, formal standardised adverse event grading scales were not prospectively applied given the exploratory nature of the study.

No clinically significant safety concerns were identified during the study period.

## 3. Discussion

This pragmatic, exploratory, real-world study assessed the potential complementary effects of oral co-supplementation with quercetin and curcumin administered alongside usual symptomatic management in individuals experiencing persistent Long COVID symptoms. The principal findings were that lower within-participant scores for symptom burden, fatigue, and pain and higher physical health-related QoL scores were observed following the 8-week study period. Numerically higher cognitive-function scores and lower anxiety, depressive-symptom, sleep-disturbance, and autonomic-symptom scores were also observed, although these changes did not reach statistical significance. Collectively, these findings provide preliminary clinical evidence to support further evaluation of polyphenol-based adjunctive interventions in adequately powered randomised controlled trials [2,3,6,7,24].

One of the most notable findings of this study was the reduction in global symptom burden assessed using the C19-YRS. Lower scores were observed across multiple C19-YRS domains at Week 8 compared with baseline, including symptom severity, functional limitation, and perceived overall health. Long COVID is characterised by a heterogeneous constellation of multisystem symptoms that can persist for months after the initial infection, often resulting in significant functional impairment and reduced QoL [1,2,3,4,5]. The approximately 36% reduction in global symptom burden score observed in this study is consistent with a potential improvement in patient-reported symptom burden; however, formally established minimal clinically important difference (MCID) thresholds for the C19-YRS in exploratory Long COVID intervention studies remain limited, and therefore these findings should be interpreted cautiously.

Fatigue represents one of the most frequently reported and disabling symptoms in Long COVID and is often described as persistent, relapsing, and disproportionate to physical activity [1,2,3,4,5]. In the present study, fatigue severity decreased consistently across multiple measurement approaches, including the FSS and the VAFS. The magnitude of reduction observed across fatigue-related measures, including the FSS and VAFS, is consistent with a potentially meaningful reduction in patient-reported fatigue; however, the clinical significance of these changes should be interpreted cautiously given the exploratory, uncontrolled design of the study. These findings are particularly relevant because fatigue has been strongly linked to persistent immune activation, mitochondrial dysfunction, and oxidative stress in post-viral syndromes [1,2,3,4,5]. Both quercetin and curcumin have been shown to modulate inflammatory signalling pathways, reduce oxidative stress, and influence mitochondrial function [9,10,12,16,18,19]. Through these mechanisms, these compounds may plausibly influence the biological processes underlying fatigue in Long COVID.

Lower pain-related scores were observed at Week 8 compared with baseline. Both pain severity and pain interference scores declined markedly over the intervention period, corresponding to lower pain intensity and lower pain interference with daily functioning. The observed reductions in pain-related scores are consistent with potentially clinically relevant reductions in patient-reported pain burden and functional interference; however, these findings require confirmation in controlled studies. Musculoskeletal pain and generalised body discomfort are commonly reported in individuals with Long COVID and may arise from persistent inflammatory responses, immune dysregulation, and neuroimmune sensitisation following viral infection [2,3,6,7,24]. Curcumin has been extensively studied for its anti-inflammatory and analgesic properties, partly mediated through inhibition of NF-κB signalling and suppression of pro-inflammatory cytokine production [12,25,26,27]. Quercetin similarly exhibits anti-inflammatory and antioxidant activities and has been shown to inhibit several inflammatory mediators involved in viral-induced inflammation [28,29,30]. The combined modulation of inflammatory and oxidative pathways by these compounds provides biological plausibility for the observed reductions in patient-reported pain-related scores, although these mechanisms were not directly evaluated in the present study [2,3,6,7,24].

Although changes in cognitive function, emotional well-being, and sleep quality did not reach statistical significance, consistent numerical changes were observed across these domains. Cognitive impairment, often referred to as “brain fog”, is a common neurological manifestation of Long COVID and has been linked to neuroinflammation, endothelial dysfunction, and microvascular injury [2,3,6,7,24]. Experimental studies suggest that both quercetin and curcumin exert neuroprotective and anti-inflammatory effects, including modulation of oxidative stress and inflammatory signalling pathways within neural tissues [31,32,33,34,35,36,37,38]. While the present study was not powered to detect modest neuropsychological changes, the observed pattern of findings supports further investigation of these domains in larger studies.

Autonomic dysfunction has also been increasingly recognised as a key feature of Long COVID, frequently presenting with symptoms such as orthostatic intolerance, gastrointestinal disturbances, and manifestations resembling postural orthostatic tachycardia syndrome (POTS) [1,2,3,4,5]. In the present study, autonomic symptom burden assessed using the COMPASS-31 scale showed numerically lower scores, particularly within the gastrointestinal domain. Although these changes did not reach statistical significance, the observed direction of change warrants further investigation in adequately powered studies. Persistent inflammation, endothelial dysfunction, and neuroimmune signalling have been proposed as potential contributors to autonomic disturbances in Long COVID [2,3,6,7]. Polyphenolic compounds such as quercetin and curcumin have been shown to modulate oxidative stress, endothelial function, and inflammatory signalling pathways, providing biological plausibility for further investigation of these compounds in autonomic manifestations of Long COVID [26,39,40].

Higher physical health-related QoL scores were observed at Week 8 compared with baseline. The PCS score of the SF-36 increased significantly after supplementation, corresponding to higher perceived physical health status. Although higher physical QoL scores were observed, interpretation of their clinical relevance should remain cautious in the absence of a comparator group and established intervention-specific Minimal Clinically Important Difference (MCID) thresholds for Long COVID populations. Although the mental component score did not reach statistical significance, exploratory multivariate analysis suggested a difference in the combined PCS–MCS QoL profile. This finding should be interpreted cautiously given the exploratory study design and small sample size and considered supportive of the univariate findings rather than independent evidence of an overall treatment effect.

The observed findings are biologically plausible given the complementary molecular actions of quercetin and curcumin. Both compounds possess anti-inflammatory, antioxidant, and immunomodulatory properties and have been shown to influence key signalling pathways implicated in COVID-19 pathogenesis, including NF-κB, MAPK, and NLRP3 inflammasome activation [9,12,26,28,29,37,39]. In addition, both compounds have been reported to reduce pro-inflammatory cytokine production and improve endothelial function, processes that are increasingly recognised as important contributors to Long COVID pathophysiology [9,12,26,28,29,37,39].

Virus-induced cellular senescence has also been reported as a potential mechanism contributing to persistent inflammation and tissue dysfunction following SARS-CoV-2 infection [13]. Virus-induced senescence can generate a senescence-associated secretory phenotype characterised by sustained release of inflammatory mediators and pro-thrombotic factors [13]. Quercetin has been identified as a potential senolytic compound capable of modulating senescence-associated inflammatory pathways, providing an additional biologically plausible mechanism through which it could influence post-viral inflammatory processes [13].

The present findings are also consistent with previous clinical studies investigating quercetin and curcumin supplementation in acute COVID-19 [10,11,16,17,18,20]. Randomised clinical trials evaluating the combination of these compounds have reported accelerated symptom resolution and earlier viral clearance in mild-to-moderate COVID-19 outpatients [21,22]. In hospitalised patients, supplementation with the same combination was associated with improved clinical condition and earlier hospital discharge compared with standard care [23]. Although these studies were conducted in the acute phase of infection, the biological mechanisms targeted by these compounds—including inflammation, oxidative stress, and endothelial dysfunction—are also implicated in the pathophysiology of Long COVID. The present exploratory study provides preliminary clinical observations supporting further investigation of this combination in the post-acute phase of SARS-CoV-2 infection. Although quercetin and curcumin have been reported in previous experimental and clinical studies to modulate inflammatory, oxidative stress, endothelial, and senescence-related pathways, the present study did not directly evaluate mechanistic biomarkers. Therefore, the proposed biological mechanisms underlying the observed clinical improvements remain hypothetical and should be interpreted cautiously.

From a clinical perspective, the findings of this study provide preliminary evidence that polyphenol-based nutritional interventions warrant further investigation as a safe and accessible complementary strategy for individuals experiencing persistent symptoms after COVID-19. Given that current Long COVID management strategies remain largely supportive and symptom-oriented, interventions capable of targeting underlying inflammatory and oxidative pathways represent a biologically plausible adjunctive approach that warrants further investigation [2,3,6,7]. Importantly, the intervention was administered alongside routine symptomatic management without modification of participants’ existing medical treatment, demonstrating the feasibility of evaluating this complementary approach in a real-world clinical setting.

Nevertheless, several limitations of this exploratory study should be acknowledged. First, the non-randomised, open-label, single-arm design without participant or investigator blinding and without a placebo or comparator group precludes causal attribution of the observed changes to the intervention, as placebo effects, spontaneous symptom variability, regression to the mean, and the natural course of Long COVID may have contributed to the findings. In addition, participants continued individualised concomitant medications and supportive therapies as part of routine clinical care. Although these treatments remained stable throughout the intervention, their potential contribution to the observed changes cannot be excluded. Second, the relatively small sample size limits statistical power and the generalisability of the results. No formal sample size calculation was performed because the study was intentionally designed as an exploratory, hypothesis-generating investigation rather than to formally evaluate efficacy. Third, although most participants were fully vaccinated against COVID-19, the study was not designed or powered to evaluate the potential influence of vaccination status on persistent symptoms or treatment response [41]. Fourth, the 8-week follow-up period may not fully capture the fluctuating and often prolonged natural history of Long COVID or the durability of the observed changes. Finally, although validated patient-reported outcome measures were used throughout the study, these remain subjective and may be influenced by recall, expectation, and reporting biases. Furthermore, objective biological markers and plasma concentrations of quercetin and curcumin were not assessed, limiting direct evaluation of the biological mechanisms underlying the observed findings and systemic exposure to the intervention. Future studies should combine validated patient-reported outcome measures with objective laboratory biomarkers to better characterise the biological effects of quercetin–curcumin co-supplementation in Long COVID. Consequently, the findings should be interpreted as preliminary and hypothesis-generating. Future adequately powered randomised, placebo-controlled trials with longer follow-up and incorporation of objective biomarkers of inflammation, oxidative stress, endothelial function, and immune activation, together with functional outcome measures, are warranted to confirm these findings and further elucidate the mechanisms of action of quercetin–curcumin co-supplementation in Long COVID.

## 4. Materials and Methods

### 4.1. Study Design and Setting

This was a single-centre, open-label, single-arm, pragmatic exploratory interventional study conducted in the outpatient clinics of Lady Reading Hospital—Medical Teaching Institution (LRH-MTI), Peshawar, Pakistan.

The study was performed between June 2025 and November 2025 in a real-world clinical practice setting. The intervention was administered as complementary therapy alongside standard of care, without modification of participants’ routine medical management. The objective was to explore the potential complementary effects of combined oral supplementation with quercetin and curcumin, administered with standard care, on persistent post-COVID-19 symptom burden in adults experiencing Long COVID.

The study protocol was approved by the Institutional Review Board (IRB), Lady Reading Hospital—MTI, Peshawar, Pakistan (Ref. No.: 267/LRH/MTI; 19 June 2025), and the Research Ethics Committee (REC), Liaquat University of Medical & Health Sciences (LUMHS), Jamshoro, Pakistan (Approval No.: LUMHS/REC/-735; 13 May 2025). The study was conducted in accordance with the principles of the Declaration of Helsinki and Good Clinical Practice (GCP) guidelines. Written informed consent was obtained from all participants prior to enrolment. The study was prospectively registered at ClinicalTrials.gov (Identifier: NCT06974058, registered on 12 May 2025).

### 4.2. Participants

Participants were recruited from outpatient clinics at Lady Reading Hospital—Medical Teaching Institution (LRH-MTI), Peshawar, Pakistan. Adults aged 18–75 years with a prior RT-PCR-confirmed history of SARS-CoV-2 infection at least 6 months before enrolment were screened for eligibility.

Inclusion criteria were (i) persistent post-COVID-19 symptoms for at least 3 months following acute infection, consistent with WHO diagnostic criteria for Long COVID [42]; (ii) mild-to-moderate overall symptom burden, defined as a baseline total score of ≥25 and ≤60 on the C19-YRS, with no individual symptom rated in the severe range (score ≥ 8); (iii) stable medical condition without hospitalisation within the preceding 3 months; and (iv) willingness and ability to comply with the study protocol, including follow-up assessments and adherence to supplementation.

Exclusion criteria included (i) known hypersensitivity or allergy to quercetin or curcumin; (ii) severe or uncontrolled chronic illness (e.g., advanced heart failure, chronic kidney disease requiring dialysis, or severe liver disease); (iii) regular use of quercetin or curcumin or other polyphenol-based dietary supplementation within the preceding 3 months or current use at the time of screening; (iv) use of anticoagulant therapy (e.g., warfarin, heparin, direct oral anticoagulants); (v) acute illness at the time of screening; (vi) pregnancy or breastfeeding; (vii) use of systemic immunosuppressive medications or corticosteroids within the past month; and (viii) significant psychiatric disorders that could interfere with study participation or compliance.

### 4.3. Supplementation

Participants received an oral nutraceutical formulation containing quercetin and turmeric extract (Nasafytol^®^, Tilman S.A., Baillonville, Belgium), administered as a complementary therapy alongside their standard of care. Each capsule contained 65 mg of bioactive quercetin and a bioactive extract of *Curcuma longa* standardised to 42 mg of curcumin. The supplementation regimen consisted of two capsules per administration, taken twice daily after meals (breakfast and dinner), providing a total daily intake of 260 mg of quercetin and 168 mg of curcumin, for 8 weeks. The selected dosing regimen was based on prior clinical experience using the same curcumin–quercetin formulation (Nasafytol^®^) in previous COVID-19 clinical studies, in which the same dosing schedule demonstrated acceptable tolerability and exploratory clinical benefits in mild-to-moderate COVID-19 patients [21,22,23]. The intervention was administered as complementary therapy alongside standard of care, without modification of participants’ routine medical management. Standard care consisted of routine symptom-directed management according to usual clinical practice, which included pharmacological treatments such as analgesics (e.g., paracetamol or non-steroidal anti-inflammatory drugs), antidepressants, sleep medications, and inhaled therapies for respiratory symptoms, as well as rehabilitation-based interventions depending on the individual patient’s symptom profile. Concomitant medications and symptom-directed therapies being used as part of routine clinical care were documented at baseline and monitored throughout the study period during follow-up assessments and weekly telephone contacts. A summary of concomitant symptom-directed therapies and supportive management used during the study period is provided in Table S1. Participants were instructed not to initiate any new nutraceutical or polyphenol-based supplements during the intervention. Adherence was estimated through participant self-report at the 8-week follow-up visit and review of supplement use and was monitored and reinforced through weekly telephone contacts during the intervention period.

### 4.4. Outcome Measures and Study Assessments

Study assessments were conducted at baseline and at Week 8. All outcome measures were collected using validated patient self-administered questionnaires during study visits.

The study’s primary endpoint was the change in overall Long COVID symptom burden from baseline to Week 8, assessed using the C19-YRS [43]. The C19-YRS is a validated patient-reported outcome measure developed specifically for post-COVID-19 syndrome, assessing symptom severity and functional impact across multiple domains, with higher total scores indicating greater symptom burden. Total and domain-specific scores (symptom severity, functional limitations, overall health, and additional symptoms) were calculated according to the original C19-YRS scoring framework, in which individual items are rated on a 0–10 numerical scale and summed to generate domain and total scores, as previously described by O’Connor et al. [43].

Secondary endpoints included changes in individual symptom domains and related patient-reported outcomes, including the following:

Fatigue was assessed using the FSS, a 9-item self-administered questionnaire evaluating the impact of fatigue on daily activities and functioning; each item is rated on a 7-point Likert scale, with higher scores indicating greater fatigue severity [44].

Pain was assessed using the BPI-SF, a validated 9-item self-administered patient-reported questionnaire that measures both pain intensity (0–10 numerical rating scale) and the degree to which pain interferes with daily activities, with higher scores indicating greater pain severity and interference [45].

Mood status (anxiety and depressive symptoms) was assessed using the HADS, a 14-item self-report tool comprising separate anxiety and depression subscales, with higher scores reflecting greater psychological symptom burden [46].

Perceived cognitive function was assessed using the PROMIS-CF-8a, an 8-item standardised patient-reported measure evaluating difficulties in memory, attention, and mental clarity, with higher T-scores indicating better perceived cognitive functioning [47].

Sleep quality was assessed using the PSQI, a validated self-administered patient-reported questionnaire that evaluates subjective sleep quality and sleep disturbances over the preceding month, with higher global scores indicating poorer sleep quality [48].

Dysautonomia-related symptoms were assessed using the COMPASS-31, a validated self-administered patient-reported questionnaire that quantifies autonomic symptom burden across multiple domains (e.g., orthostatic intolerance, gastrointestinal, vasomotor, secretomotor, bladder, and pupillomotor symptoms), with higher total scores indicating greater autonomic symptom severity [49].

Health-related QoL was assessed using the SF-36, which generates physical and mental health component scores, with higher scores representing better perceived health status [50].

Supplement tolerability and safety were assessed through participant-reported adverse events at the Week 8 follow-up visit and during weekly telephone contacts throughout the intervention period. No objective laboratory safety measurements were performed.

### 4.5. Statistical Analysis

Given the exploratory nature of the study, the relatively small sample size, and the potential non-normal distribution of patient-reported outcome measures, non-parametric statistical methods were used throughout. Continuous variables are presented as median (interquartile range [IQR]) and categorical variables as numbers (%). Paired comparisons between baseline and Week 8 were performed using the Wilcoxon signed-rank test. To account for multiple comparisons, *p*-values were adjusted using the Benjamini–Hochberg false discovery rate (FDR) procedure, with adjusted *p*-values reported as q-values. Effect sizes for paired Wilcoxon analyses were calculated as *r* = |*Z*|/√*N*, where *Z* is the standardised Wilcoxon statistic, and *N* is the number of paired observations, and were interpreted descriptively given the exploratory single-arm design.

Health-related QoL was assessed using both univariate and multivariate approaches. Changes in the SF-36 Physical Component Summary (PCS) and Mental Component Summary (MCS) scores were evaluated using paired Wilcoxon tests. An exploratory PERMANOVA based on Euclidean distance and 9999 permutations while accounting for within-subject pairing was performed to assess overall changes in the combined PCS–MCS profile. Given the exploratory design and small sample size, PERMANOVA findings were considered supportive of the univariate analyses and should be interpreted cautiously.

PROMIS-CF-8a raw scores were converted to standardised T-scores according to published scoring algorithms and normative reference values [51,52]. SF-36 domain and component summary scores were calculated and normalised using the *lbscorer* R package [53]. All tests were two-sided, with FDR-adjusted *q* < 0.05 considered statistically significant, whereas values of 0.05 ≤ *q* < 0.10 were interpreted descriptively as non-significant trends. Statistical analyses and graphical visualisations were performed in R (version 4.5.0) [54], using the *ggplot2* [55], *microbAIDeR* [56], and *vegan* [57] packages.

### 4.6. Sample Size Considerations

Given the exploratory nature of the study, no formal sample size calculation was performed. A sample of 15 participants meeting eligibility criteria during the study period was enrolled. The study was designed to generate preliminary data and estimate effect sizes to inform future, adequately powered randomised controlled trials.

## 5. Conclusions

This exploratory, real-world clinical study provides preliminary evidence that oral co-supplementation with quercetin and curcumin warrants further investigation as a potentially useful complementary supportive strategy in adequately powered randomised controlled trials. Supplementation was associated with lower patient-reported overall symptom burden, fatigue, and pain scores, together with improved physical health-related QoL within this uncontrolled exploratory study. However, these treatment-associated findings should be interpreted cautiously and should not be considered evidence of efficacy, given the uncontrolled exploratory study design, until confirmed in adequately powered randomised placebo-controlled trials. If confirmed, these findings may support the potential role of polyphenol-based interventions as part of a multimodal therapeutic approach for Long COVID.

## Figures and Tables

**Table 1:** Baseline clinical characteristics of the study population (*n* = 15).

Characteristic	Overall ^1^
Age (years)	37.00 [20.00]
Weight (kg)	69.00 [12.50]
Body mass index (kg/m^2^)	24.60 [10.35]
Systolic blood pressure (mmHg)	130.00 [7.50]
Diastolic blood pressure (mmHg)	85.00 [9.00]
Heart rate (bpm)	80.00 [5.50]
Respiratory rate (breaths/min)	15.00 [1.00]
Body temperature (°C)	36.70 [0.30]
Year of acute COVID-19 infection	
2021	10 (67%)
2022	4 (27%)
2023	1 (6.7%)
Acute COVID-19 severity	
Home-Based Recovery	14 (93%)
Hospitalised	1 (6.7%)
Time to Long COVID onset	
3 months post-infection	4 (27%)
4 months post-infection	5 (33%)
5 months post-infection	2 (13%)
6 months post-infection	4 (27%)
Comorbidity	
No	10 (67%)
Yes	5 (33%)
COVID-19 vaccination status	
Fully vaccinated	14 (93%)
Not Vaccinated	1 (6.7%)

^1^ Data are presented as median (interquartile range [IQR]) for continuous variables and *n* (%) for categorical variables.

## Data Availability

The original contributions presented in this study are included in this article. Further inquiries can be directed to the corresponding author.

## Supplementary Materials

The following supporting information can be downloaded at: https://www.mdpi.com/article/10.3390/ph19081202/s1, Table S1: Concomitant symptom-directed therapies and supportive management during the study period (*n* = 15). Figure S1: Longitudinal changes in the remaining autonomic symptom domains assessed using the self-reported Composite Autonomic Symptom Score (COMPASS-31) following 8 weeks of supplementation.
