# Supplementary material for: Potential Adjunctive Effects of Quercetin–Curcumin Co-Supplementation in Adults with Mild-to-Moderate Long COVID: Results from a Pragmatic Exploratory Real-World Clinical Study
Source: Pharmaceuticals (Basel). 2026 Jul 31;19(8):1202. doi: 10.3390/ph19081202 (PMC13516766; doi:10.3390/ph19081202)
Supplement: Supplementary file 1 [file pharmaceuticals-19-01202-s001.zip › pharmaceuticals-4449000-supplementary.pdf]

Table S1. Concomitant symptom-directed therapies and supportive management during the study period (*n* = 15).

Category of therapy	Medication	Participants using therapy, n (%)
Analgesics/anti-inflammatory therapies	Paracetamol, NSAIDs	15 (100)
Antidepressants/anxiolytics	SSRIs, anxiolytics	10 (66.7)
Sleep medications	Sedative/hypnotic therapy	9 (60.0)
Respiratory therapies	Inhaled corticosteroids	6 (40.0)
Rehabilitation/lifestyle interventions	Physical activity, breathing exercises	15 (100)
No concomitant pharmacological therapy	—	0 (0)

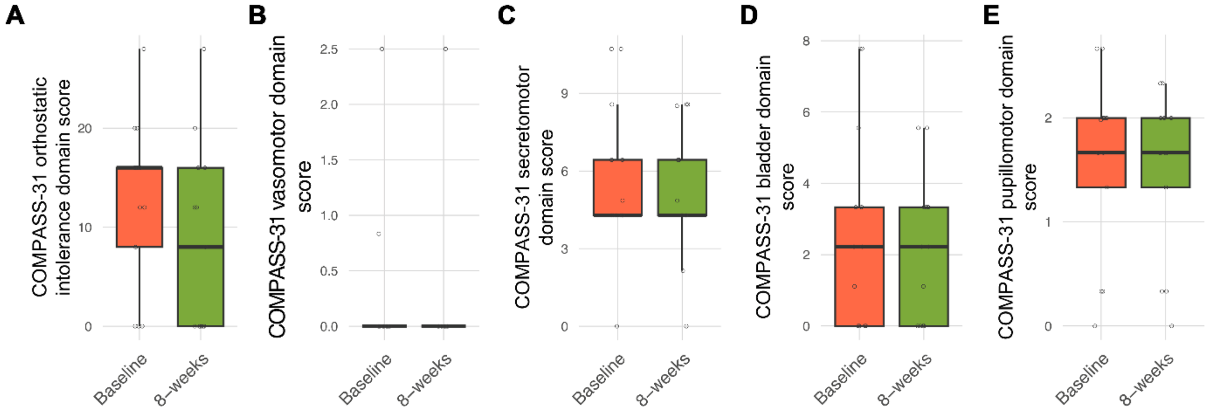

Figure S1. Longitudinal changes in the remaining autonomic symptom domains assessed using the self-reported Composite Autonomic Symptom Score (COMPASS-31) following 8 weeks of supplementation. Panels show the COMPASS-31 domain scores for (A) orthostatic intolerance, (B) vasomotor symptoms, (C) secretomotor symptoms, (D) bladder symptoms, and (E) pupillomotor symptoms. Boxes represent the interquartile range (IQR), center lines indicate the median, whiskers extend to 1.5 × IQR, and points represent individual participants. Paired comparisons between baseline and Week 8 were performed using Wilcoxon signed-rank tests with FDR correction. No statistically significant changes were observed in the domains shown.
